# Effect of vitamin D_3_ supplementation on cardiometabolic disease risk among overweight/obese adult males in the UK: A pilot randomised controlled trial

**DOI:** 10.1111/jhn.13021

**Published:** 2022-05-09

**Authors:** Tarimoboere Agbalalah, Sohail Mushtaq

**Affiliations:** ^1^ Department of Biological Sciences Baze University Abuja Nigeria; ^2^ Department of Clinical Sciences and Nutrition University of Chester Chester UK

**Keywords:** cardiometabolic disease, endothelial function, inflammation, obesity, randomised, vitamin D_3_

## Abstract

**Background:**

Observational studies suggest links between reduced serum 25(OH)D concentration and increased cardiometabolic disease risk. However, these studies provide limited evidence of causation, with few conclusive randomised controlled trials (RCT) having been carried out to date. This RCT investigated the effect of vitamin D_3_ supplementation on vascular function and cardiometabolic disease risk markers, in 55 healthy males aged 18–65 years with plasma 25(OH)D concentration <75 mol L^–1^ and body mass index ≥24.9 kg m^–2^.

**Methods:**

Participants were assigned to consume 125 µg day^–1^ (5000 IU day^–1^) vitamin D_3_ or placebo for 8 weeks. Blood samples and vascular function measures were obtained at baseline, as well as at weeks 4 and 8. The primary outcome was arterial stiffness, an indicator of cardiovascular disease (CVD) risk, assessed by pulse wave velocity. Biomarkers of CVD risk, insulin resistance and endothelial function were measured using an enzyme‐linked immunosorbent assay.

**Results:**

Daily oral intake of 125 µg supplemental vitamin D_3_ led to a significant improvement in plasma 25(OH)D concentrations over the 8‐week intervention in the vitamin D group compared to the change in the placebo group (*p* *˂* 0.001). In the vitamin D group, the baseline mean ± SD 25(OH)D concentration was 38.4 ± 15.9 and this increased to 72.8 ± 16.1 nmol L^–1^ after 8 weeks of supplementation. The intervention had no effect on arterial stiffness, as measured by pulse wave velocity, although vitamin D_3_ supplementation did lead to a decrease in mean ± SD brachial pulse pressure from baseline to 8 weeks of −2.9 ± 3.4 mmHg (*p* = 0.027) in the vitamin D group compared to the same period in the placebo group. The intervention had no effect on the remaining cardiometabolic parameters.

**Conclusions:**

Overall, treatment significantly improved brachial pulse pressure but no other cardiometabolic disease risk markers. To follow on from this pilot RCT, future large‐scale clinical trials over longer durations may offer further insights.

## Key points

- This pilot randomised controlled trial investigated the effect of vitamin D_3_ on arterial stiffness, biomarkers of cardiovascular disease risk and insulin resistance in vitamin D insufficient overweight and obese adult males.
- Supplementation significantly decreased brachial pulse pressure in the vitamin D_3_ group at 4 and 8 weeks but did not improve the other cardiometabolic markers measured.
- The findings from the present study are in agreement with previous research that does not support the use of daily high‐dose vitamin D supplements for the purpose of reducing risk of cardiovascular disease.

## Graphical abstract

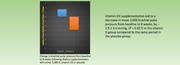

## INTRODUCTION

Observational studies have described correlations between vitamin D deficiency as indicated by low serum 25(OH)D status and cardiometabolic disease risk factors,^1^, ^2^ including endothelial dysfunction,^3^ inflammation,^4^ insulin resistance,^5^ hypertension,^6^ dyslipidaemia,^7^ oxidative stress^8^ and arterial stiffness.^9^ These relationships are supported by evidence which shows that many pathways and cell types implicated in cardiovascular disease (CVD) pathogenesis are regulated through vitamin D metabolites because most cardiovascular and inflammatory cells express vitamin D receptor (VDR) and CYP27B1, a mitochondrial 1α‐hydroxylase enzyme that catalyses the conversion of inactive vitamin D to its active form.^10^

For beneficial extra‐skeletal outcomes, Bischoff–Ferrari suggested improving serum concentrations of 25(OH)D to 75–100 nmol L^–1^.^11^ However, much of this evidence is established by observational studies that are unable to provide strong evidence of causality. The limited number of randomised controlled trials (RCT) evaluating the effect of supplemental vitamin D on cardiometabolic disease risk factors, particularly endothelial function and arterial stiffness in various human populations, are inconsistent. A metanalysis of 81 RCTs found that vitamin D supplementation significantly reduced systolic and diastolic blood pressure (SBP and DBP), high‐sensitivity C‐reactive protein (hs‐CRP) total cholesterol (TC) low‐density lipoprotein cholesterol (LDL‐C), triacylglycerols (TAG) and significantly increased high‐density lipoprotein cholesterol (HDL‐C), but did not significantly impact arterial stiffness parameters.^11^ Some of the studies included in this meta‐analysis, recorded significant reductions in SBP and DBP following vitamin D supplementation, in overweight and obese participants, who are the target group in our study. However, the number of studies investigating arterial stiffness measures were limited and inconclusive, indicating a need to carry out further studies incorporating arterial stiffness measures. Additionally, some of the studies included in the meta‐analysis administered vitamin D along with calcium, which is a confounding factor. Furthermore, participants of some of these RCTs were older, with already established cardiovascular disease.^12^ However, in contrast to these findings, meta‐analyses of RCTs^13^, ^14^ and further individual vitamin D supplementation studies^15^, ^16^, ^17^ did not provide conclusive evidence on the beneficial effects of vitamin D on cardiovascular outcomes.

The possible mechanisms by which optimal vitamin D influences vasoprotection may be stimulation of the production of endothelial nitric oxide,^18^ downregulation of the renin–angiotensin system^19^ and modulation of the inflammatory processes and lipid metabolism.^20^ Vitamin D may also directly regulate vascular smooth muscle cell production^21^ and inhibit the harmful effects of advanced glycation end‐products on vascular ageing.^22^

In addition to low serum 25(OH)D concentrations, adiposity has been implicated in the pathogenesis of cardiometabolic disease risk.^23^ Inverse associations have been described between low serum 25(OH)D_3_ and adiposity, possibly as a result of the dilution of ingested or cutaneously synthesised vitamin D in the enlarged fat mass.^24^

Inconsistent findings have been reported by RCTs evaluating the effect of supplemental vitamin D on cardiometabolic risk, and these trials were often powered on non‐cardiometabolic outcomes. The aim of this pilot RCT was to investigate the effect of a daily oral intake of 125 µg of vitamin D_3_ on haemodynamic measures, including arterial stiffness, insulin resistance and biomarkers of CVD risk in vitamin D‐insufficient overweight and obese adult males.

## METHODS

### Study population

Healthy overweight/obese adult males aged 18–65 years with body mass index (BMI) ≥ 24.9 kg m^–2^ and plasma 25(OH)D concentration <75 nmol L^–1^ were recruited using study posters/leaflets and newspaper advertisements. The threshold was selected as 75 nmol L^–1^ and was regarded as the upper threshold for insufficiency.^25^ Participants were excluded if they had previously been clinically diagnosed with cardiometabolic, renal, liver or gastrointestinal disease and were taking supplemental vitamin D. Participants provided written informed consent, and trial was performed in accordance with the Helsinki Declaration.^26^ Consolidated Standards of Reporting Trials (CONSORT) were also followed.^27^ Ethical approval was obtained from the University of Chester Faculty Research Ethics Committee (REF: 855/13/AT/CSN) and the trial was registered at clinicaltrials.gov (NCT02359214). The study was conducted from November 2014 to May 2015 and from October 2015 to January 2016 aiming to reduce the impact of UVB radiation exposure from the sun. Although four participants finished in early May 2015, their final vitamin D concentration was not significantly different from the overall group.

### Study design

The trial was a randomised, double‐blinded and placebo‐controlled. Participants were randomly allocated to an 8‐week intervention with oral vitamin D_3_ supplements, containing 125 µg of cholecalciferol, calcium phosphate, microcrystalline cellulose, magnesium stearate and silica or a placebo containing lactose taken daily. The dose of 125 µg day^–1^ (5000 IU day^–1^) of vitamin D_3_ was selected because it was shown to increase plasma 25(OH)D levels by approximately 220% in a 12‐week intervention in 30 patients with serum 25(OH)D ≤ 50 nmol L^–1^, with a mean post‐intervention plasma 25(OH)D concentration of 114.4 ± 22.2 nmol L^–1^.^28^ Although the present RCT duration is 8 weeks, the post‐intervention value from the study is significantly higher than the optimal threshold of 75 nmol L^–1^, and a significant increase was thus deemed achievable in the 8‐week timeframe of the present study. The dose used in the present study has been safely used in previous intervention studies (including pregnant women) and it has been shown to significantly increase serum/plasma 25(OH)D concentration.^28^ Vitamin D_3_ and placebo tablets were purchased from Bulk Powders and Placebo‐world, respectively, and were indistinguishable for blinding purposes. The investigator, participants and research staff were blinded to study allocation until the trial was completed. A third party assigned the participants to either the vitamin D_3_ or the placebo group by means of a computer‐generated random number sequence (www.randomization.com). Block randomisation was utilised to ensure balance in sample size across the groups. The third party was also responsible for packaging tablets into tamper‐proof containers, and sealing them in sequentially numbered study packs.

Compliance was estimated by counting unused tablets in the containers at the conclusion of study using: % compliance =  (actual/expected) × 100.

### Dietary intake

Participants' dietary intake over three successive days (2 weekdays and 1 weekend day) was evaluated using a 3‐day food diary and was completed at baseline and the final week of the study (week 8). Food and drink intake between meals or at night were noted. Participants recorded all fortified foods, and for homemade dishes, the recipe, quantity of ingredients and cooking method were documented in the diary. Mean daily energy, protein, fat, carbohydrate and vitamin D intake were assessed using dietary analysis software (Nutritics, version 4.25; Nutritics).^29^

### Measurements

All participants at the screening clinic, received a participant information sheet, and were asked to complete a screening questionnaire as well as an informed consent document. Participants' BMI was obtained by measuring weight and height using calibrated scales and a stadiometer. To confirm eligibility, 1 ml of blood drawn from the median cubital vein was used to assess vitamin D status. Participants with plasma 25(OH)D concentrations below 75 nmol/L were invited to take part in the study. At baseline, as well as the 4‐ and 8‐week clinics, following an overnight fast, venous blood samples were collected from each participant to assay 25(OH)D, parathyroid hormone (PTH), LDL‐C, HDL‐C, TC, non‐HDL‐C, TAG, hs‐CRP, sE‐selectin, renin, angiotensin II, glucose, insulin and 8‐isoprostane concentrations. To avoid clotting, venous blood was drawn with a 21‐gauge vacutainer needle into sterile 10‐ml lithium heparin and EDTA tubes and stored at 4°C. Plasma was separated by centrifuging whole blood for 10 min at 4°C at 2054 *g* and aliquoted into microcentrifuge tubes and stored at −80°C until batch analysis, with the exception of the screening clinic samples, for which 25(OH)D concentrations were determined within 24 h, aiming to ascertain whether the participant was eligible for the study. Overall, participants attended four clinics (screening, baseline, weeks 4 and 8).

### Measurement of plasma vitamin D concentration

Plasma concentration of 25(OH)D was measured with a VIDAS® 25(OH)D total assay kit (BioMẻrieux), which applies the enzyme linked fluorescent assay method on the mini VIDAS® automated immunoassay‐analyser. The intra‐ and interassay coefficients of variation (CV) were 2.0% and 7.3%, respectively. For plasma samples with lower 25(OH)D concentrations (below 20.3 nmol L^–1^) that the automated immuno‐analyser was unable to detect, an enzyme‐linked immunosorbent assay (ELISA) (Calbiotech) was used.

### Biomarkers

Plasma sE‐selectin (collected in heparin tubes) and renin concentration were assessed using ELISA kits (R&D Systems Europe). Intra‐ and interassay CV for plasma sE‐selectin and renin concentration were 3.4% and 3.4% and 8.9 % and 5.3%, respectively. Plasma PTH, hs‐CRP and insulin concentrations were determined using an ELISA (Calbiotech). Intra‐ and interassay CV were 4.7% and 2.6%; 6.0% and 2.3%; and 3.4% and 8.9%, respectively. Plasma glucose and TC concentrations were measured by means of a colorimetric enzyme reagent kit (Alpha Laboratories). Intra‐ and interassay CV for plasma glucose and TC were 3.0% and 5.3% and 5.2% and 6.4%, respectively. The plasma 8‐isoprostane concentration was determined using a competitive in vitro ELISA (Abcam). The intra‐ and interassay CV was 5.9% and 11.1%, respectively. Plasma TAG was measured using a quantitative enzymatic TAG determination kit (TRO100; Sigma‐Aldrich). HDL‐C was determined using a HDL quantitation kit in which HDL is first precipitated and then the cholesterol concentration is determined by a coupled enzyme assay, resulting in a colorimetric product (MAK045‐1KT; Sigma‐Aldrich). Plasma, angiotensin II concentration were determined using a competitive enzyme immunoassay kit (RAB0010; Sigma‐Aldrich). Intra‐ and interassay CV for TAG, HDL‐C and angiotensin II were 2.6% and 6.2%; 3.4% and 8.1%; and 9.5% and 8.9 %, respectively.

LDL‐C concentration was determined by means of the Friedewald formula^30^:

Plasma LDL‐C = Plasma TC − Plasma HDL‐C − (TRG/2.2).

Non‐HDL‐C concentration was determined using: non‐HDL‐C = TC − HDL‐C.

Insulin resistance and homeostasis model assessment of insulin resistance (HOMA‐IR) was using: HOMA‐IR =  (fasting plasma glucose concentration (mmol L^–1^) × fasting plasma insulin concentration (mU L^–1^)/22.5.^31^

### Arterial stiffness

Prior to fasted venous blood samples being drawn at baseline, as well as 4‐ and 8‐week clinics, measurement of arterial function parameters was carried out in a quiet room at 22 ± 1°C, with the participant consuming a glass of water and then lying in a supine position for 10 min before a cuff was placed firmly around the right arm. Arterial function parameters determined, include aortic pulse wave velocity (PWVao), brachial and aortic augmentation indices (AIx), central systolic blood pressure (SBPao), return time of aortic pulse wave (RTao), SBP and DBP, heart rate (HR), mean arterial pressure (MAP), brachial pulse pressure (PP) and central aortic pulse pressure (PPao), using a non‐invasive clinically validated automatic oscillometric device (Arteriograph 5‐01, version 1.9; TensioMed). Aortic distance was obtained as the distance between jugular notch and symphysis pubis (Jug‐Sy) using a measuring tape with participant standing upright. For each participant, three measurements, each lasting 2–3 min were performed with the average of the last two readings being documented. The SD of the PWVao was checked to inform the investigator about the quality of the measurement. When the SD for PWVao was ≥0 and ≤1.0 m s^–1^, the measurement was regarded to be of good quality. However, measurement was rejected and repeated when SD PWVao was ≥ 1.0 m s^–1^.

Each measurement was performed in accordance with the protocols of the Arteriograph device (Tensiomed).^32^

The PWVao and both aortic and brachial AIX were measured using the Arteriogaph with the formulas^32^:

PWVao (m/s) = Jug‐Sy (m)/(RT/2 (s)

AIx (%) = (P2 − P1)/PP) × 100

### Statistical analysis

Continuous variables were assessed for normality and homogenous variance at baseline using Shapiro–Wilk and Levene's test, respectively. Student's independent *t* test or a Mann–Whitney *U* test was used to assess the difference between groups for all normally and nonnormally distributed baseline outcomes, respectively. Descriptive statistics were represented as the mean ± SD.

To evaluate the interaction between treatment groups and time on parameters measured, mixed model repeated measures analysis of variance (ANOVA) was performed on continuous variables that met assumptions of normality, homogenous variance and sphericity (when sphericity was violated one of the epsilon correction factors (Greenhouse‐Geisser) was consulted. Continuous variables that showed statistically significant interactions between groups at different time points were further analysed by performing a follow‐up test, which, in this case, comprise a multiple independent *t* test with Bonferroni adjustment to avoid a type 1 error.

To evaluate interaction between treatment groups and time on parameters that did not meet assumptions of mixed model repeated measures ANOVA at all time points, a Mann–Whitney *U* test was conducted.

For continuous variable that were normally distributed with a homogeneous variance at either baseline, or at weeks 4 or 8, an independent *t* test was conducted to evaluate the interaction between groups and time.

Data were analysed using SPSS, version 22 (IBM Corp.). *p* ˂ 0.05 was considered statistically significant. As this RCT is a pilot study, no sample size estimation was conducted; however, the outcomes of this study will be of use to researchers who wish to carry out post‐hoc sample size estimations.

## RESULTS

**Figure 1:**
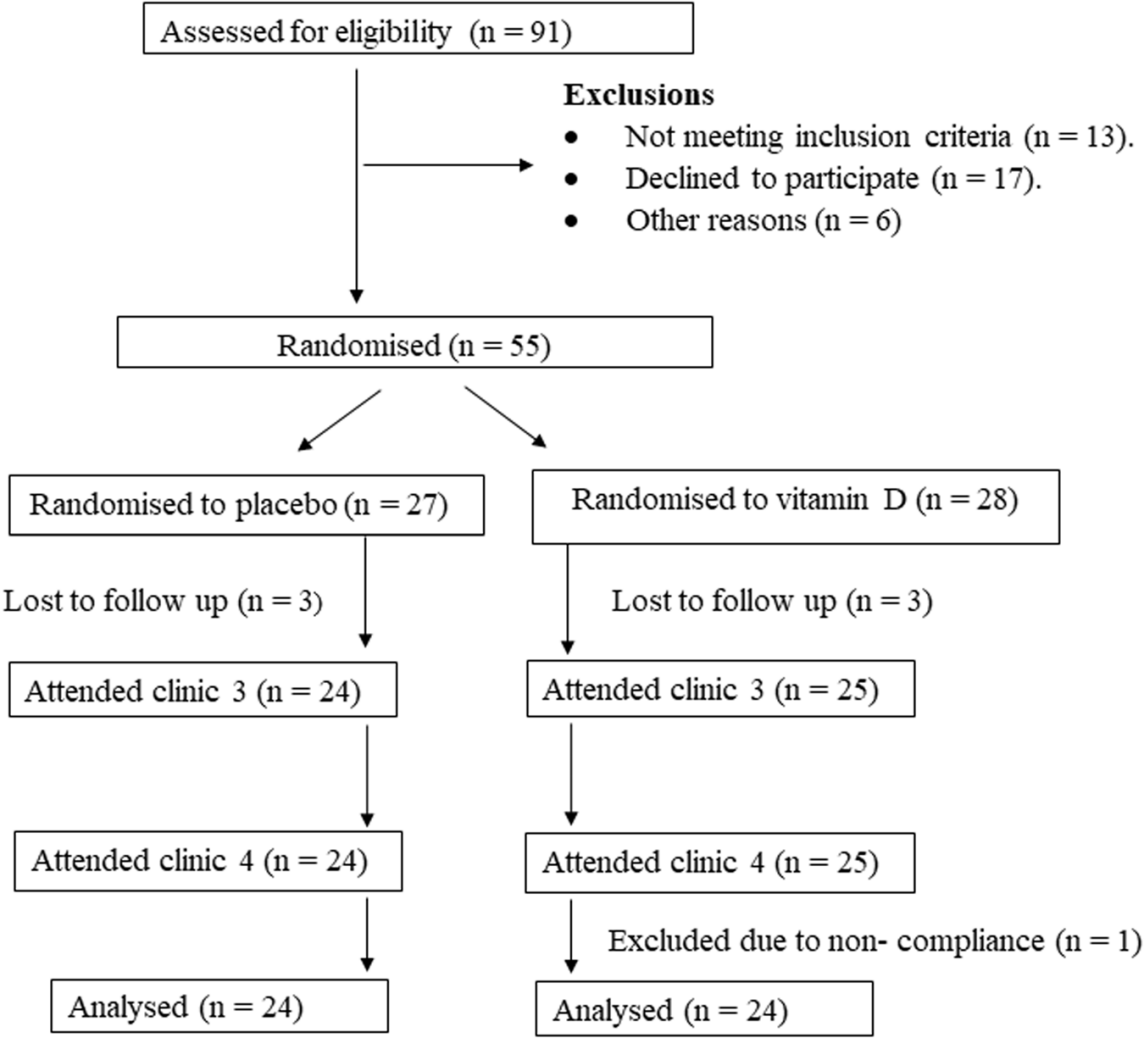
Flow chart of study population.

Ninety‐one participants were screened for eligibility and 55 males were assigned to the intervention (Figure 1 **)**. The compliance rate was 90% in the vitamin D group and 87% in the placebo group. No adverse events of supplementation were reported.

Mean ± SD age was 35.9 ± 11.8 years in the vitamin D group and 33.1 ± 12.2 years in the placebo group. Participant characteristics at baseline in the different intervention groups are presented in Table 1 **.** No significant differences at baseline were observed in the variables measured between groups, except for the vitamin D group having a slightly higher mean ± SD plasma glucose 5.23 ± 1.45 vs. 4.75 ± 0.82 ± mmol L^–1^ (*p* = 0.025) compared to the placebo group. At baseline, 22.2 % (*n* = 12), 35.2 % (*n* = 19) and 64.8% (*n* = 35) of participants had plasma 25(OH)D concentrations ˂25, ˂30 and ˂50 nmol L^–1^, respectively, independent of treatment group.

**Table 1:** Baseline characteristics of the study population in the vitamin D_3_ and placebo group.

Parameters	Vitamin D_3_	Placebo	*p*
Plasma 25(OH)D (nmol L^–1^)	38.5 ± 16	44.0 ± 19.5	0.544
			
Plasma PTH (pmol L^–1^)	4.3 ± 3.5	4.2 ± 2.8	0.917
*Haemodynamic measures*			
SBP (mmHg)	128.7 ± 11.1	131.2 ± 12.8	0.730
DBP (mmHg)	77.0 ± 9.7	78.0 ± 12.9	0.797
PWV (m s^–1^)	6.5 ± 1.1	6.5 ± 1.1	0.802
PP (mmHg)	53.6 ± 9.2	53.8 ± 9.2	0.846
MAP (mmHg)	95.1 ± 9.1	95.9 ± 12.0	0.796
AIx (brachial) (%)	−48.6 ± 25.9	−51.5 ± 22.2	0.953
AIxao (aortic) (%)	12.6 ± 13.4	12.7 ± 13.6	0.940
SBPao (mmHg)	118.5 ± 12.9	119.5 ± 16.9	0.873
PPao (mmHg)	41.6 ± 8.8	41.3 ± 8.7	0.927
HR (bpm)	62.3 ± 12.3	60.9 ± 10.8	0.635
RT (m s^–1^)	153.3 ± 24.4	157.6 ± 24.9	0.443
*Cardiometabolic markers*			
Plasma soluble E‐selectin (ng ml^–1^)	57.9 ± 31.1	57.5 ± 18.9	0.312
Plasma hs‐CRP (mg L^–1^)	2.9 ± 1.9	2.8 ± 2.1	0.480
Plasma 8‐isoprostane (pg ml^–1^)	11.2 ± 8.7	8.9 ± 5.7	0.449
Plasma renin (pg ml^–1^)	639.8 ± 294	695.1 ± 410.7	0.716
Plasma angiotensin II (pg ml^–1^)	32.3 ± 9.6	31.8 ± 9.8	0.810
Plasma insulin (pmol L^–1^)	46.5 ± 27.7	42.1 ± 37.0	0.160
HOMA‐IR	1.6 ± 1.3	1.4 ± 1.6	0.068
Plasma glucose (mmol L^–1^)	5.2 ± 1.5	4.8 ± 0.8	0.025
*Blood lipids*			
Plasma TC (mmol L^–1^)	6.5 ± 1.8	6.4 ± 1.6	0.758
Plasma TAG (mmol L^–1^)	1.7 ± 0.4	1.7 ± 0.3	0.665
Non‐HDL‐C (mmol L^–1^)	6.3 ± 1.8	6.2 ± 2.2	0.893
Plasma HDL‐C (mmol L^–1^)	0.6 ± 0.1	0.7 ± 0.2	0.814
LDL‐C (mmol L^–1^)	5.2 ± 1.5	5.0 ± 1.6	0.719
*Anthropometry/dietary intake*			
Age (years)	35.9 ± 11.8	33.1 ± 12.2	0.248
Body weight (kg)	92.4 ± 10.3	90.4 ± 19.3	0.378
Body mass index (kg m^–2^)	29.9 ± 3.3	28.4 ± 2.6	0.071
Waist circumference (cm)	93.5 ± 22	91.3 ± 8.0	0.085
Energy (kcal day^–1^)	1853.9 ± 500.8	2141.7 ± 663.8	0.194
Energy (MJ day^–1^)	7.5 ± 2.2	8.8 ± 2.9	0.110
Fat (g day^–1^)	68.4 ±14.2	82.0± 27.4	0.062
Protein (g day^–1^)	83.9 ± 24.5	106.9 ± 56.9	0.147
Carbohydrate (g day^–1^)	219.4 ± 67.5	247.8 ± 85.1	0.251
Vitamin D (g day^–1^)	1.6 ± 1.2	3.1 ± 3.1	0.060

Values are presented as the mean ± SD.

* *p <* 0.05

Abbreviations: 25(OH)D, 25 hydroxyvitamin D; AIx, aortic augmentation index; AIxao, brachial augmentation index; DBP, diastolic blood pressure; HDL‐C, high density lipoprotein cholesterol; HOMA‐IR, homeostasis model assessment of insulin resistance; HR, heart rate; hs‐ CRP, high sensitivity C‐reactive protein; LDL‐C, low density lipoprotein cholesterol; MAP, mean arterial pressure; non‐HDL‐C, non‐ high density lipoprotein cholesterol; PP, brachial pulse pressure; PPao, central aortic pulse pressure; PTH, parathyroid hormone, PWV, pulse wave velocity; RT, return time; SBP, systolic blood pressure; SBPao, central systolic blood pressure; sE‐selectin, soluble E‐selectin and TC, total cholesterol; TAG, triacylglycerol.

Based upon reported dietary consumption from the completed 3‐day food diary at weeks 0 and 8 (*n* = 42), no significant difference was observed between the intervention and placebo group in mean daily dietary intake of energy, carbohydrate, protein, fats and vitamin D. Baseline dietary intake is presented in Table 1.

**Figure 2:**
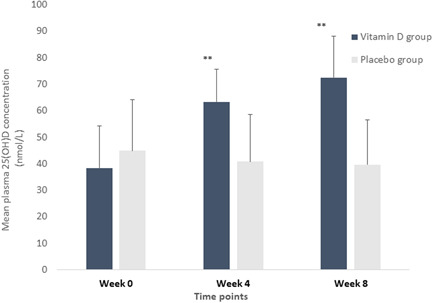
Plasma 25(OH)D concentrations at each timepoint during the 8‐week intervention in the vitamin D and placebo groups. Values are presented as the mean ± SD indicated as vertical error bars. Asterisks above error bars denote a statistically significant within‐group difference in plasma 25(OH)D concentration at that time point compared to baseline (week 0), analysed using the Mann–Whitney *U* test (*p* ˂ 0.001).

Daily intake of 125 µg of vitamin D_3_ within the vitamin D group, improved plasma 25(OH)D concentrations significantly, from a baseline mean ± SD concentration of 38.5 ± 16.0 to 62.5 ± 19.5 nmol L^–1^ (*p* *˂* 0.001) at 4 weeks and 72.5 ± 16.8 nmol L^–1^ (*p ˂* 0.001) at 8 weeks **(**Figure 2 **)**. Participants in the vitamin D_3_ group, 45.8% (11/24) reached a plasma 25(OH)D concentration >75 nmol L^–1^ after 8 weeks of supplementation.

The key finding of the RCT was that Vitamin D_3_ supplementation led to a decrease in mean ± SD brachial pulse pressure from baseline to 8 weeksof −2.9 ± 3.4 mmHg, (*p* = 0.027) in the vitamin D group compared to the same period in the placebo group. The intervention had no effect on the remaining parameters (Table 2).

**Table 2:** Effect of 8 weeks vitamin D_3_ supplementation on cardiometabolic disease risk markers.

	Vitamin D_3_ (*n* = 24)			Placebo (*n* = 24)			
Parameters	Baseline	Week 4	Week 8	Baseline	Week 4	Week 8	*p*
Plasma 25(OH)D (nmol L^–1^)	38.5 ± 16.0	62.5 ± 19.5	72.5 ± 15.8	44.0 ± 19.5	39.0 ± 17.5	38.8± 18.0	< 0.001***
Plasma PTH (pmol L^–1^)	4.3 ± 3.5	4.5 ± 3.5	3.7 ± 3.0	4.2 ± 2.8	4.4 ± 2.8	4.9 ± 3.1	0.112
*Haemodynamic measures*							
SBP (mmHg)	128.7 ± 11.1	126.7 ± 11.9	127.8 ± 11.0	131.2 ± 12.8	134.1 ± 12.9	135.5 ± 13.0	0.099
DBP (mmHg)	77.0 ± 9.7	76.0 ± 8.8	77.0 ± 9.0	78.0 ± 12.9	79.3 ± 13.0	80.1 ± 13.4	0.522
PWV (m s^–1^)	6.5 ± 1.1	6.4 ± 1.0	6.4 ± 0.8	6.5 ± 1.1	6.3 ± 0.9	6.3 ± 0.9	0.423
PP (mmHg)	53.6 ± 9.2	49.4 ± 3.9	50.7 ± 5.8	53.8 ± 9.2	53.5 ± 6.4	55.4 ± 9.7	0.027*
MAP (mmHg)	95.1 ± 9.1	93.4 ± 8.2	94.2 ± 8.2	95.9 ± 12.0	96.0 ± 12.4	97.2 ± 12.7	0.413
AIx (brachial) (%)	−48.6 ± 25.9	−42.8 ± 24.8	−33.2 ± 12.6	−51.5 ± 22.2	−45.5 ± 13.1	−45.4 ± 13.3	0.940
AIxao (aortic) (%)	12.6 ± 13.4	16.1 ± 13.9	15.7 ± 14.1	12.7 ± 13.6	11.5 ± 9.6	12.5 ± 16.8	0.705
SBPao (mmHg)	118.5 ± 12.9	116.9 ± 12.6	117.9 ± 12.7	119.5 ± 16.9	119.4 ± 14.8	121.4 ± 18.7	0.347
PPao (mmHg)	41.6 ± 8.8	40.5 ± 7.5	40.9 ± 7.3	41.3 ± 8.7	41.4 ± 10.8	43.1 ± 10.5	0.280
HR (bpm)	62.3 ± 12.3	61.2 ± 11.7	61.7 ± 11.7	60.9 ± 10.8	62.0 ± 11.6	60.9 ± 11.0	0.904
RT (m s^–1^)	153.3 ± 24.4	153.9 ± 26.4	154.4 ± 28.1	157.6 ± 24.9	162.9 ± 27.6	159.5 ± 27.3	0.715
*Cardiometabolic markers*							
Plasma soluble E‐selectin (ng ml^–1^)	57.9 ± 31.1	44.2 ± 29.4	47.1 ± 29.7	57.5 ± 18.9	39.2 ± 17.6	40.4 ± 16.0	0.733
Plasma hs‐CRP (mg L^–1^)	2.9 ± 1.9	2.4 ± 1.7	2.7 ± 1.4	2.8 ± 2.1	3.1 ± 2.1	2.6 ± 2.1	0.264
Plasma 8‐isoprostanes (pg ml^–1^)	11.2 ± 8.7	16.5 ± 11.1	14.6 ± 11.5	8.9 ± 5.7	15.8 ± 12.5	16.4 ± 13.2	0.222
Plasma renin (pg ml^–1^)	639.8 ± 294	559.9 ± 253.8	463.6 ± 244.5	695.1 ± 410.7	542.2 ± 243.3	471.6 ± 186.5	0.610
Plasma angiotensin II (pg ml^–1^)	32.3 ± 9.6	31.0 ± 6.7	29.5 ± 4.6	31.8 ± 9.8	32.7 ± 12.5	27.9 ± 7.3	0.390
Plasma insulin (pmol L^–1^)	46.5 ± 27.7	43.6 ± 26.9	46.0 ± 17.3	42.1 ± 37.0	48.3 ± 42.4	46.6 ± 39.9	0.897
HOMA‐IR	1.6 ± 1.3	1.6 ± 1.3	1.7 ± 2.1	1.4 ± 1.6	1.4 ± 2.1	1.4 ± 1.9	0.680
Plasma glucose (mmol L^–1^)	5.2 ± 1.5	5.0 ± 1.4	5.3 ± 2.1	4.8 ± 0.8	4.5 ± 0.7	4.8 ± 0.5	0.209
*Blood lipids*							
Plasma TC (mmol L^–1^)	6.2 ± 1.3	6.8 ± 1.6	7.5 ± 1.5	6.4 ± 1.6	6.8 ± 2.0	7.3 ± 1.7	0.216
Plasma TAG (mmol L^–1^)	1.7 ± 0.4	1.6 ± 0.4	1.7 ± 0.5	1.7 ± 0.3	1.6 ± 0.3	1.6 ± 0.2	0.445
Non‐ HDL‐C (mmol L^–1^)	6.3 ± 1.8	5.6 ± 1.1	6.4 ± 2.7	6.2 ± 2.2	5.6 ± 1.8	6.2 ± 1.5	0.740
Plasma HDL‐C (mmol L^–1^)	0.6 ± 0.1	0.6 ± 0.1	0.7 ± 0.1	0.7 ± 0.2	0.6 ± 0.1	0.6 ± 0.1	0.250
LDL –C (mmol L^–1^)	5.2 ± 1.5	5.6 ± 1.5	5.4 ± 2.4	5.0 ± 1.6	5.5 ± 1.6	5.5 ± 1.0	0.416

Values are presented as the mean ± SD. The *p* value represents the significance level for the change in parameters from baseline to week 8 in the vitamin D group compared to the change in the same parameter in the placebo group. Mixed model repeated measures analysis of variance was performed to determine the effect of the intervention.

## DISCUSSION

This pilot RCT, investigated the effect of daily dietary oral supplementation with 125 µg of vitamin D_3_ for 8 weeks in overweight and obese adult males on a comprehensive array of cardiometabolic risk markers, including endothelial function, arterial stiffness, oxidative stress and insulin resistance. Vitamin D_3_ supplementation significantly increased plasma levels of 25(OH)D after 8 weeks but did not significantly improve the cardiometabolic markers evaluated between intervention groups. Nevertheless, favourable effects were found in brachial PP because vitamin D_3_ supplementation led to a decrease in mean ± SD brachial PP from baseline to 8 weeks of −2.9 ± 3.4 mmHg (*p* = 0.027) in the vitamin D group compared to the same period in the placebo group.

Although a previous systematic review and meta‐analysis of nine RCTs reported significant improvement in arterial stiffness following supplementation in vitamin D deficient adults,^33^ the findings of the present study are consistent with results from other studies that reported no significant effect of supplemental vitamin D on cardiometabolic markers.^34^, ^35^ A narrative review evaluating the effectiveness of vitamin D supplementation in 45 RCTs of various patient populations reported that no RCTs were effective in reducing stiffness in large arteries, or improving atherosclerotic and endothelial function markers with or without vitamin D deficiency.^33^ Additionally, a RCT in 35 healthy participants with 25(OH)D concentrations below 75 nmol L^–1^ found that 12 weeks of administration of 50 µg of vitamin D did not alter lipid profile, fasting plasma, insulin and hs‐CRP concentrations.^35^

Even though plasma 25(OH)D concentration significantly improved in the vitamin D_3_ group in the present study, the majority of participants were unable to attain the 75 nmol L^–1^ threshold. This could be attributed to adipose sequestration in the overweight/obese participants, as reports in humans demonstrate that approximately 17% of vitamin D given orally is stored in adipose tissue.^36^ Furthermore, those with darker skin may have to supplement with higher doses to achieve optimal levels of plasma 25(OH)D than used in the present study.^37^ Another possible reason for inability of some participants in the vitamin D_3_ group to reach plasma 25(OH)D concentrations above 75 nmol L^–1^ may be the decreased expression of 25‐hydroxylase CYP2J and 1‐α hydroxylase CYP27B1 in adiposity.^38^ Additionally, it could also be ascribed to circulating vitamin D binding protein (VDBP) because VDBP has a greater binding affinity for 25(OH)D compared to 1,25(OH)_2_D^39^, ^40^ and levels of VDBP are reported to be reduced in acute inflammation, suggesting that, in obesity, which is characterised by chronic low‐grade inflammation, circulating VDBP levels may be reduced.^41^, ^42^ Furthermore, VDR genotype polymorphisms particularly the VDR *ff* genotype, has been described to exhibit a low response to vitamin D intake and is also associated with adiposity.^43^, ^44^, ^45^ Consequently, VDR genotype polymorphism in adiposity could be an additional explanation for the reduced individual response to a high dose of vitamin D_3_ in the present study.

The impact of VDR genotype polymorphism that has been revealed to influence individual response to vitamin D intake in obese individuals was not evaluated in present study.^41^ Assessing individual VDR genotype polymorphism in people with varying adiposity, particularly in the overweight and obese could be vital to understanding variability in individual response to oral intake of vitamin D.^43^ However, few RCTs in overweight and obese participants have investigated the impact of VDR genotype polymorphisms on plasma concentrations of 25(OH)D.

The absence of a beneficial impact of high dose vitamin D_3_ supplementation on cardiometabolic risk markers in this study could be attributed to a number of factors. First of all, a higher dose and extended duration of supplementation with vitamin D_3_ may perhaps be needed, particularly in overweight/obese participants, because plasma 25(OH)D concentrations ˃ 75 nmol L^–1^ are needed for optimum extra‐skeletal health.^11^

In the present study, enrolment of healthy participants with no known pathology at baseline may be a likely reason for the lack of changes observed in SBP, DBP and MAP compared to an 8‐week Iranian study in hypertensive and vitamin D deficient outpatients.^46^ The study revealed significant reductions in mean ± SD SBP (−6.4 ± 5.3 vs. 0.9 ± 3.7 mmHg, *p* < 0.001), DBP (−2.4 ± 3.7 vs. 1.0 ± 2.7 mmHg, *p* = 0.003) and MAP (−3.7 ± 3.6 vs. 0.9 ± 2.5 mmHg, *p* < 0.001) in the vitamin D compared to placebo group, following an 8‐week intake of 1250 µg vitamin D_3_.^44^ The study found no significant impact of vitamin D_3_ supplementation on pulse pressure.^46^ In the absence of changes in blood pressure measurements, the change in brachial PP in the present study may be less impactful because brachial PP reflects changes in peripheral arteries and not in large conduit arteries, and is less effective than SBP or DBP in the predictive value of CVD risk.^47^

Additionally, using non‐diabetic participants at baseline could be another potential reason for the absence of significant changes in insulin resistance and plasma levels of insulin and glucose. It is likely that supplementation with vitamin D_3_ could be favourable in insulin resistant people, as significant reductions and improvement were observed in fasting insulin and insulin resistance respectively, following 6 months intake of 100 µg of vitamin D_3_ in insulin resistant South Asian women who were vitamin D deficient (25(OH)D < 50 nmol L^–1^).^48^ This RCT found a decrease in insulin resistance at a serum 25(OH)D concentration of 80–119 nmol L^–1^, showing a dose response relationship between vitamin D concentrations and insulin resistance.^48^

Most of the participants in the present study possibly have less established anatomical changes in their arterial tree because they were physically active and below the age of 50 years. Cardiometabolic risk increases with age, and only few participants above 50 years were recruited; thus, the ability to determine significant differences in this age group was limited. It is possible that vitamin D has differential effects dependent on specific cardiometabolic outcomes.^49^

Finally, it should be noted that the present study was a pilot intervention and, as such, there is a possibility that it was underpowered, and due to the large number of outcome variables there is a risk of type 1 error. Because there was no change in the primary outcome measure of PWV, the resulting effect size is low (0.12). Therefore, a post‐hoc power calculation (two tailed) with 80% power and 0.5% significance level estimates a required sample size of 1140 participants per group (G*Power, version 3.1.9.7; http://www.gpower.hhu.de).

The present study has a number of strengths, such as the randomised double‐blind, placebo‐controlled design, as well as a strong and reliable evidence of a treatment effectiveness that permits causal inferences to be drawn.^50^ The present study also evaluated a number of cardiometabolic markers at various time points, which, over time, are capable of tracking an effect, and the study also controls for influences that induce variations between subjects.^51^

In summary, supplementation with an 8‐week daily dose of 125 µg of vitamin D_3_ in overweight and obese adult males did not lead to a significant improvement in the cardiometabolic markers measured. However, in the vitamin D group, PP significantly decreased from baseline to 8 weeks compared to the change in the placebo group. Overall, the findings of this pilot RCT did not demonstrate the efficacy of vitamin D supplementation in improving cardiometabolic risk biomarkers in the cohort of overweight/obese adults over the 8‐week duration. Further large‐scale clinical trials over longer durations may offer additional insights.

## AUTHOR CONTRIBUTIONS

Tarimoboere Agbalalah drafted the study and manuscript under the supervision of Sohail Mushtaq. All authors reviewed and approved the final version of the manuscript submitted for publication.

## CONFLICTS OF INTEREST

The authors declare that there are no conflicts of interest.

## CLINICAL TRIAL REGISTRATION

http://www.clinicaltrials.gov NCT02359214.

## TRANSPARENCY DECLARATION

The lead author affirms that this manuscript is an honest, accurate and transparent account of the study being reported. The reporting of this work is compliant with CONSORT^25^ guidelines. The lead author affirms no important aspect of the study was omitted and that any discrepancies from the study as planned have been explained.

## ETHICS STATEMENT

The author affirms that all participants provided written informed consent and trial was performed in accordance with the Helsinki Declaration. Author affirms ethical approval was obtained for all participants and trial was registered at clinicaltrials.gov (NCT02359214).
